# Mechanisms for successful management of enterprise resource planning from user information processing and system quality perspective

**DOI:** 10.1038/s41598-023-39787-y

**Published:** 2023-08-04

**Authors:** Hyeon Jo, Do-Hyung Park

**Affiliations:** 1 Headquarters, HJ Institute of Technology and Management, 71 Jungdong-ro 39 104-1602, Bucheon-si, Gyeonggi-do, Bucheon, 14721 Republic of Korea; 2 Graduate School of Business IT, Kookmin University, 77 Jeongneung-ro, Seongbuk-gu, Seoul, 02707 Republic of Korea

**Keywords:** Psychology, Mathematics and computing

## Abstract

Enterprise resource planning (ERP) systems are now ubiquitous in modern organizations. A number of previous studies have focused only on system factors and perceptions, there is a noticeable shortfall in research that concurrently addresses technological factors and human roles in explaining user satisfaction. This study aimed to identify these variables from the perspectives of information systems, technology, and human participation, thereby addressing this knowledge gap. The focus of the study was a large shipbuilding and marine company utilizing an ERP system. The participants, a sample of 234 ERP users, were carefully selected by the company’s executives and practitioners, and data was collected through online questionnaires. They were selected through purposive sampling from among employees who use ERP systems in large-scale shipbuilding and marine engineering companies. The study aimed to clarify the relationships between user satisfaction and perceived ease of use, perceived usefulness, system quality, service quality, participation, and information quality. A partial least squares structural equation modeling (PLS-SEM) was used to analyze the collected data. The results indicated that perceived ease of use, system quality, service quality, and participation positively influenced user satisfaction, whereas perceived usefulness did not have a significant impact. Interestingly, participation was found to lessen the effects of perceived usefulness on satisfaction. The findings of this study suggest that to enhance ERP user satisfaction, managers should strive to make the ERP system easy-to-use and stable, encourage employee participation in the decision-making process, and bolster the role of the support team. It should be noted, however, that the study has limitations as it did not consider all possible factors, such as training and support. Future research should take a broader view of the variables involved in the operation of an enterprise-wide information system.

## Introduction

Information technology (IT) offers numerous benefits, such as cost-saving^1^, customer satisfaction^2^, and production flexibility^3^ to firms for managing processes^4^. Particularly, most large and medium organizations have adopted enterprise resource planning (ERP) systems to increase organizational effectiveness^5^. ERP systems represent comprehensive software packages designed to integrate all corporate operations and processes, offering a complete view from a single IT architecture^6^. They support key corporate operations including manufacturing, supply chain management, and human resources, among others^7^. Most companies operate an ERP system because it allows managers to access real-time statistics on sales, profitability, and inventory levels^8^. An increasing number of organizations have begun to agree that they need a modern ERP system to keep up with their competitors^9^. With a market value of $35.81 billion in 2018 and an expected $78.4 billion in 2026, ERP system utilization is still increasing^10^.

User satisfaction is a critical determinant of ERP system success^11^. A satisfied user is likely to accept and use the system more effectively, thereby improving organizational efficiency and productivity^12^. Over the years, numerous studies have explored the factors influencing ERP user satisfaction, focusing on aspects such as system quality, information quality, and service quality^13^. Despite the extensive research on ERP user satisfaction, there are notable gaps in the literature. Many studies have treated user satisfaction as a unidimensional construct, neglecting the reality that satisfaction is multifaceted and influenced by a combination of technological and human factors^14^. Furthermore, the influence of context-specific factors such as organizational culture or industry type on ERP user satisfaction has been largely overlooked in previous research^15^. Given these gaps in the literature, there is a clear need for a more comprehensive exploration of the factors influencing ERP user satisfaction. Research is needed to elucidate the relationships between user satisfaction and understudied variables like technological factors, perception elements, and human engagement.

The quality of an ERP system is a determinant of its usability and reliability^16^. High system quality ensures that the ERP system functions smoothly, with minimal errors or breakdowns, enabling users to complete their tasks efficiently^17^. Conversely, poor system quality can lead to user frustration and dissatisfaction, as it impedes their ability to perform their work effectively^18^. The quality of the information provided by the ERP system is vital for decision-making processes within an organization^19^. Information that is accurate, timely, relevant, and complete enhances user satisfaction as it allows them to make informed decisions and perform their tasks more effectively^20^. Conversely, poor information quality can lead to incorrect decisions and inefficiencies, resulting in user dissatisfaction. The quality of the services provided to ERP system users, such as technical support and training, can greatly influence user satisfaction. High service quality ensures that users receive the necessary support to use the system effectively, which in turn enhances their satisfaction and acceptance of the system^21^. On the other hand, poor service quality can lead to user frustration and dissatisfaction. Thus, understanding the effects of system, information, and service quality on user satisfaction is key to improving service practices and user support in ERP system implementations.

Perceived ease of use and perceived usefulness are critical to user satisfaction with ERP systems. If a system is perceived as easy to use, it reduces the cognitive load on the user, increasing satisfaction^22^. Conversely, a system perceived as difficult to use can lead to dissatisfaction. If users perceive the system as useful and believe it can improve their job performance, they are more likely to be satisfied with the system^23^. Understanding the relationship between these factors and user satisfaction can inform strategies to enhance user satisfaction.

User participation in the development and implementation of a system is crucial for several reasons. Firstly, users who participate in system development and implementation are more likely to have a better understanding of the system’s functionalities and are thus better equipped to use the system effectively^24^. Secondly, participation can enhance users’ sense of ownership and commitment to the system, leading to higher satisfaction^25^. Lastly, user participation can ensure that the system is designed to meet users’ needs, thus enhancing its perceived usefulness and ease of use^26^.

Moreover, the role of user participation can extend beyond a direct influence on satisfaction; it may also moderate the effects of perceived ease of use, perceived usefulness, and service quality on satisfaction. For instance, when users participate in system development, they may perceive the system as easier to use because they are familiar with its design and functionalities, thereby enhancing their satisfaction^27^. Similarly, user participation may enhance perceived usefulness, as users can ensure that the system is tailored to their needs, thus increasing their satisfaction. Furthermore, user participation may also influence the relationship between service quality and satisfaction. When users are actively involved in the implementation process, they may have more direct communication with service providers, leading to a better perception of service quality and, consequently, higher satisfaction^28^. Given the potential moderating role of user participation, it is crucial to consider this factor in studies on ERP user satisfaction. Such considerations can provide insights into how to enhance user satisfaction by fostering user participation.

The study is underpinned by several fundamental theories related to user satisfaction and technology adoption. The technology acceptance model (TAM), developed by Davis^12^, is one of the most influential models for understanding user acceptance and usage behavior of information systems (ISs). It posits that perceived usefulness and perceived ease of use are the two primary determinants of user acceptance of a system. In the context of this study, TAM helps to explain how these factors can influence ERP user satisfaction. Moreover, a part of this study is based on the DeLone and McLean (D&M) ISs success model^11^. This model suggests that ISs success can be evaluated based on six interrelated dimensions: system quality, information quality, service quality, use, user satisfaction, and net benefits. This study incorporates the concepts of system quality, information quality, and service quality from this model and explores their relationship with user satisfaction in the context of ERP systems. Finally, this paper borrows core logics from the user participation theory^25^. This theory posits that user participation in system development and implementation can lead to higher user satisfaction^25^. It provides the basis for considering user participation as a determinant of ERP user satisfaction and as a potential moderator in the relationships between other variables and satisfaction. By integrating these theories, this study offers a comprehensive theoretical framework for understanding ERP user satisfaction, considering a range of technological and human factors.

Despite the extensive body of research on ERP systems, there remain several gaps in our understanding of the factors that influence user satisfaction, leading to knowledge lags in this area. Primarily, while individual studies have examined factors such as system quality, information quality, service quality, perceived ease of use, perceived usefulness, and user participation separately, few have considered these factors collectively within a single study. This means we have a limited understanding of the interrelationships between these variables and their collective impact on user satisfaction. Secondly, although user participation is widely recognized as important, its potential moderating effect on the relationships between other variables and satisfaction has not been thoroughly explored. The role of user participation, particularly as a moderator, is thus still not well-understood. Lastly, there has been a strong focus on the adoption of ERP systems in previous research, with less attention given to user satisfaction, especially in the post-implementation stage. This has resulted in a knowledge lag regarding the factors that can enhance user satisfaction after ERP systems have been implemented. Considering these knowledge lags, the objective of this study is threefold:

1. To provide an integrative understanding of the determinants of ERP user satisfaction by examining system quality, information quality, service quality, perceived ease of use, perceived usefulness, and user participation collectively.
2. To explore the potential moderating role of user participation in the relationships between perceived ease of use, perceived usefulness, service quality, and user satisfaction.
3. To focus on user satisfaction in the post-implementation stage of ERP systems, providing insights that can help organizations enhance the effectiveness of their ERP systems after implementation.

**Figure 1:**
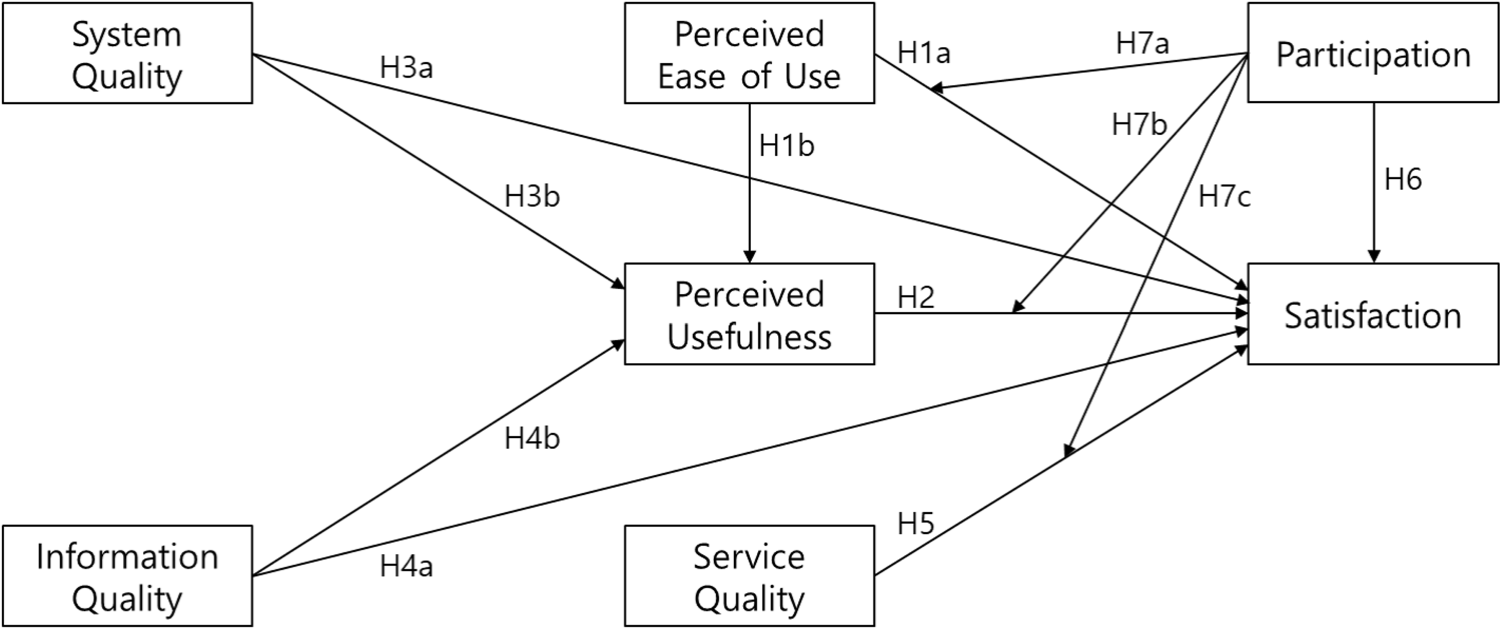
Research model.

To achieve these objectives, this study suggests a conceptual framework as displayed in Fig. 1. The study posits that perceived ease of use affects satisfaction directly and indirectly via perceived usefulness. This paper also postulates that system quality and information quality influence perceived usefulness and satisfaction. Moreover, it proposes that service quality and user participation affect satisfaction. Finally, the current study hypothesizes that participation moderates the effects of perceived ease of use, perceived usefulness, and service quality on satisfaction.

This paper is organized in a structured manner. It begins with a literature review in “Literature review and hypotheses formulation” section, focusing on ERP, and introducing the hypotheses. The research methodology is explained in “Research methodology” section. The results of the data analysis are presented in “Empirical results” section. In “Discussion” section, the findings are discussed. “Results” section outlines the theoretical and practical implications of the study, along with a discussion on the limitations of the research and future directions for further investigation.

## Literature review and hypotheses formulation

The extant literature on user satisfaction with ERP systems is expansive and diverse. A significant portion of this literature has utilized quantitative research methodologies to examine the relationships between various constructs and ERP user satisfaction. While our study follows this quantitative tradition, we acknowledge the insightful critiques and perspectives provided by qualitative research in this area. To facilitate a more comprehensive understanding of our study’s relevance and its theoretical contributions, the study begins by reviewing the primary models and theories relevant to this investigation, followed by a thorough examination of empirical studies pertaining to ERP. Ultimately, this process guides the establishment of hypotheses.

The D&M IS success model was first presented in 1992 by DeLone and McLean to describe the success of ISs^29^. According to the model, system quality and information quality of ISs determine use and user satisfaction, and affect individual and organizational impact. Later, the authors updated the framework by adding service quality to the model and replacing the impacts with net benefits^11^. Researchers have extensively applied and validated the D&M model in multiple IS contexts^30–34^. In early research, much of the focus was on the technical aspects of ERP systems, examining factors such as system quality, system integration, and customization capabilities^35–37^. For example, Li and Zhu^38^ unveiled that quality of e-learning system and information quality influence user satisfaction. Koksalmis and Damar^39^ discovered the significant impacts of consultant support perceived ease of use in the case of ERP. Cheng et al.^40^ confirmed that system quality and information quality are significant factors that influence perceived usefulness and satisfaction. Andreas and Natariasari^41^ argued that system quality and service quality are important factors that contribute to the satisfaction of ERP users. Ghani et al.^33^ demonstrated that system quality and service quality have a positive impact on the performance of employees using ERP.

TAM explains user behaviors in the process of technology acceptance^12^. According to the model, external factors determine attitude through perceived usefulness and perceived ease of use. In turn, attitude determines the use of the system through the intention to use it. IS literature has introduced TAM to explicate the behaviors of ERP users^42–44^. For instance, Uddin et al.^45^ showed that effort expectancy (similar concept to perceived ease of use) and performance expectancy (similar concept to perceived usefulness) affects actual use of ERP via intention to use. AlBar and Hoque^46^ revealed that information and communication technology (ICT) skills and ICT infrastructure determine the intention to adopt ERP. Koksalmis and Damar^39^ uncovered that perceived ease of use affects behavioral intention to use ERP indirectly through perceived usefulness. Cheng^47^ found that as users perceive ERP to be more useful, their satisfaction increases. Furthermore, Cheng^48^ confirmed that perceived ease of use indirectly influences satisfaction through perceived usefulness. This body of work provided valuable insights into the technical determinants of ERP user satisfaction and formed the foundation for our understanding of ERP success. However, this stream of research largely employed quantitative methodologies and often overlooked the nuanced human and organizational factors that influence ERP satisfaction.

Building on these qualitative insights, recent research has begun to take a more integrative approach, combining both technical and human-organizational factors in the study of ERP user satisfaction^49,50^. This line of research recognizes that while technical factors are crucial, they do not operate in a vacuum. Instead, they interact with a host of human and organizational factors to influence user satisfaction. User participation in the development and implementation of IS has been a central topic of discussion in IS research for decades, and a significant body of literature exists on this topic^51–54^. User participation in IS development is generally regarded as beneficial, with numerous studies finding a positive relationship between user participation and various measures of IS success^55–58^. User participation can improve the quality of the system design, enhance system acceptance, increase user satisfaction, and ultimately lead to more successful system usage^20,26,59,60^. For example, Boudreau and Robey^61^ used a qualitative case study approach to explore the role of emotions in ERP implementation. Their work shed light on the dynamic, emotional, and often contentious process of ERP implementation, highlighting the importance of considering the human side of ERP systems. Wu and Wang^35^ employed a mixed-methods approach, integrating qualitative and quantitative data to measure ERP success from the viewpoint of key users. Their work emphasized the importance of user perspectives in understanding the overall success of an ERP system. Matende and Ogao^54^ shed light on the importance of user participation in ERP implementation, emphasizing the need for active involvement to ensure successful outcomes. AlBar and Hoque^46^ validated the significance of top management support in influencing ERP adoption intention, highlighting the crucial role of human participation. Vargas and Comuzzi^62^ compiled a list of critical success factors for ERP implementation and included end user involvement as one of the key factors.

In summary, our study is situated within this integrative research tradition. Drawing from both the D&M IS success model and the TAM, we examine a range of both technical and human-organizational factors that may influence ERP user satisfaction. Furthermore, in response to calls for more process-oriented research on ERP systems^63^, we also consider the role of user participation—a process factor—in shaping user satisfaction. By integrating insights from these diverse research streams, we aim to provide a more holistic and nuanced understanding of the determinants of ERP user satisfaction. This approach not only allows us to address gaps in the existing literature but also positions our study at the forefront of current empirical work in this area.

### Perceived ease of use

Perceived ease of use is defined as the degree to which a user anticipates that using a particular technology will be effort-free^12^. It has been demonstrated in multiple studies that perceived ease of use can significantly enhance satisfaction with ERP systems^43,64^. This concept is intrinsically tied to the user experience, with systems that are easy to navigate and understand typically resulting in higher user satisfaction. The influence of perceived ease of use extends to perceived usefulness, with several studies demonstrating a positive correlation between these two factors^42,65–68^. This relationship is particularly significant in the context of ERP systems. ERP systems, due to their extensive functionalities, are generally more complex than other individual unit-of-work ISs. Hence, to facilitate user understanding and navigation, it is crucial that these systems are designed with an easy-to-use structure^8,69,70^. The easier an ERP system is to use, the more likely users are to be satisfied with it. Furthermore, users are also likely to perceive the ERP system as more useful when it is easy to navigate and understand. In summary, perceived ease of use plays a critical role in shaping user satisfaction and perceived usefulness, particularly in the context of complex ERP systems. As such, this study proposes the following hypotheses:

#### Hypothesis 1a

Perceived ease of use significantly influences satisfaction.

#### Hypothesis 1b

Perceived ease of use significantly influences perceived usefulness.

### Perceived usefulness

Perceived usefulness is defined as the degree of conviction a user has in the potential benefits that a specific technology can offer^12^. This factor has been shown to play a significant role in enhancing user satisfaction^71–73^, and has been identified as a critical determinant of satisfaction among ERP users^13,74^. The influence of perceived usefulness extends beyond mere user satisfaction. Research indicates that perceived usefulness can shape behavioral intention through arousing interest, especially in the context of ERP^69^. Zviran et al.^23^ further underscored that perceived usefulness boosts employer satisfaction with ERP usage, suggesting a broader organizational impact. Klaus and Changchit^8^ highlighted that perceived usefulness influences satisfaction related to task completion via attitudes toward ERP usage, reinforcing the centrality of this construct in the ERP context. Considering the above, the importance of perceived usefulness in shaping user satisfaction and other behavioral outcomes cannot be overstated. As such, this study proposes the following hypothesis:

#### Hypothesis 2

Perceived usefulness significantly influences satisfaction.

### System quality

System quality is a widely used measure to assess the effectiveness of ISs and is primarily evaluated based on functionality, stability, and usability^11,75^. The influence of system quality on user satisfaction is substantial and has been consistently confirmed across various studies^76–79^. Chaveesuk and Hongsuwan^4^ highlighted that system quality is a crucial determinant of user satisfaction. Further, the role of system quality in shaping perceived usefulness has been underscored in multiple IS contexts^80–82^. When an ERP system delivers high quality—being stable, functional, and user-friendly—users are likely to express higher satisfaction. Moreover, the more reliable and stable the ERP system, the more users perceive it to be useful. In consideration of these findings, this study puts forth the following hypotheses:

#### Hypothesis 3a

System quality significantly influences satisfaction.

#### Hypothesis 3b

System quality significantly influences perceived usefulness.

### Information quality

Information quality is a critical aspect of assessing the effectiveness of ISs in achieving their intended goals^71^. It encompasses attributes such as accuracy, clarity, and adequacy^29^. A number of studies have provided evidence that information quality directly influences user satisfaction in the context of ISs^11,77,78^. Additionally, information quality has been shown to contribute to perceived usefulness, particularly in the context of ERP systems^64^. When users perceive the information provided by an ERP system to be accurate, clear, and sufficient, they are likely to find the system more satisfying to use. This enhanced satisfaction is also likely to enhance their perception of the system’s usefulness. Hence, the following hypotheses are proposed:

#### Hypothesis 4a

Information quality significantly influences satisfaction.

#### Hypothesis 4b

Information quality significantly influences perceived usefulness.

### Service quality

Service quality in the context of IS, such as ERP, pertains to the support provided by the service provider, which could include technical support, training, and user documentation^11^. Service quality has been identified as a key determinant of user satisfaction in various IS studies^83–86^. In an ERP system, service quality refers to the extent to which the system can meet users’ needs and provide a satisfactory user experience, and this can be influenced by factors such as system reliability, responsiveness of the service provider, and the adequacy of system documentation and training. High service quality implies that the system is reliable, the service provider is responsive to users’ needs, and users are provided with adequate documentation and training to use the system effectively. A study conducted by Hsu et al.^7^ on the determinants of user satisfaction in the context of ERP systems found that service quality has a significant influence on user satisfaction. The author argued that users’ perception of service quality influences their satisfaction with the ERP system, and this is consistent with the findings of previous studies^30,31^. Similarly, a study by Lu et al.^87^ on the success factors of ERP systems identified service quality as one of the key determinants of user satisfaction. Furthermore, a study by Hsu et al.^88^ on the impact of service quality on user satisfaction in the context of ERP systems found that service quality has a positive and significant influence on user satisfaction. The authors argued that the quality of service provided by the ERP system influences users’ perception of the system’s usefulness and ease of use, which in turn affects their satisfaction with the system. Based on these findings, service quality plays a critical role in determining user satisfaction in the context of ERP systems. Therefore, the following hypothesis is proposed:

#### Hypothesis 5

Service quality significantly influences satisfaction.

### Participation

User participation represents the actions and behaviors in which users participate during the IS establishment phase^52^. Research has been conducted on how to best define and measure user participation. For instance, Barki and Hartwick^25^ proposed that user participation should be measured along three dimensions: the degree to which users are involved in the decision-making processes related to IS development, the extent of user responsibility in the development process, and the frequency of user-developer interaction. User participation is a proximal condition for the successful implementation of IT/IS^25,53,89,90^. More recent studies have focused on the role of user participation in Agile IS development methods. These methods, such as Scrum and Extreme Programming, emphasize frequent interaction between developers and users, and they view user participation as vital for successful system development^91,92^. Empirical studies on Agile methods have generally found positive effects of user participation on project outcomes, further reinforcing the importance of user participation in IS development. This multi-dimensional view of user participation provides a more nuanced understanding of how users can contribute to IS development and implementation. Users become to sense ownership when they engage in system development^93^. ERP is a system for efficiently managing tasks across the enterprise. The participation and involvement of end-users would be essential from the initial stage to the operation stage. Users with a higher level of participation would be satisfied with ERP more. Thus, this study suggests that:

#### Hypothesis 6

Participation significantly influences satisfaction.

User participation in system development and implementation has been identified as a significant determinant of IS success^59,94^. The presents study investigates the moderating effects of user participation on the relationship among variables. The role of user participation as a moderator in the relationship between perceived ease of use, perceived usefulness, service quality, and satisfaction has its roots in several theoretical and empirical works. The underlying principle is that involving users in the development process fosters a sense of ownership and familiarity with the system, which can lead to higher satisfaction and improved system use^95^. In the context of perceived ease of use, user participation can enhance users’ familiarity with the system and its functionalities, thereby making the system easier to use^27^. When users are involved in the system design and implementation, they are more likely to understand how to use the system effectively, which reduces the perceived complexity and increases the perceived ease of use, leading to higher satisfaction^56^. Regarding perceived usefulness, user participation can ensure that the system is tailored to meet users’ needs, thus enhancing its perceived usefulness^26^. When users are directly involved in system development, they can provide valuable input on what features and functionalities would be most useful for their tasks, thereby improving the perceived usefulness and, consequently, user satisfaction^96^. In terms of service quality, user participation may enhance users’ perception of the quality of services provided. When users are actively involved in the implementation process, they may have more direct communication with service providers. This direct interaction can lead to a better understanding of the system and the services provided, resulting in a higher perception of service quality and, consequently, higher satisfaction^28^. User participation can therefore moderate the relationships between perceived ease of use, perceived usefulness, service quality, and satisfaction. By involving users in the development and implementation of the IS, organizations can enhance these key determinants of satisfaction, leading to more successful IS outcomes.

#### Hypothesis 7a

Participation significantly moderates the effects of perceived ease of use on satisfaction.

#### Hypothesis 7b

Participation significantly moderates the effects of perceived usefulness on satisfaction.

#### Hypothesis 7c

Participation significantly moderates the effects of service quality on satisfaction.

## Research methodology

This research was performed in accordance with the Declaration of Helsinki.

### Instrument development

A quantitative analytical survey was employed in this study. This paper uses quantitative methodologies to test research models and hypotheses. Indicators were sourced from the existing literature to measure constructs. The measurement items were adjusted to fit the ERP framework. Specifically, this study replaced the subject of survey items in source studies with ERP. For example, it replaced “The information provided by our employee portal is understandable.” in Urbach et al.^97^ with “The information provided by ERP is understandable”. The same amendments were made to the other indicators. A seven-point Likert scale (1 = strongly disagree, 7 = strongly agree) was applied to evaluate each item. The questionnaire was first designed in English by the authors, the official business language. An expert linguist translated the original document from English to Korean. Subsequently, a meticulous review of this translated text was performed to confirm accurate conveyance of the original content. This involved a side-by-side comparison of the original and translated texts, with adjustments made as necessary. To verify the translation’s quality, the Korean questionnaire was then translated back into English by a professional translator. In the final step, the authors and the translator scrutinized the translated document to certify smooth readability and the absence of grammatical errors or awkward expressions. Furthermore, a panel of experts in IS and quantitative analysis reviewed the questionnaire for relevance, clarity, and comprehensiveness. Before distributing the questionnaire, we conducted a pretest with a small sample of ERP users to ensure clarity, relevance, and comprehensibility of the items. Based on the feedback received, we made necessary modifications to the wording and order of the items. The pretest also allowed us to check the reliability of the scales, which was confirmed as all Cronbach’s alpha values were above the commonly accepted threshold of 0.7^98^. Table S1 provides a list of the indicators and sources.

### Sample

The analytical model was validated by analyzing data collected from an online survey. While this research primarily employs a quantitative approach, elements of grounded theory have informed the interpretative aspects of the study, particularly in understanding the latent social patterns and structures that govern ERP user satisfaction. Grounded theory, as articulated by Strauss and Corbin^99^, is a research methodology that enables the development of theory through the systematic gathering and analysis of data. It is particularly suited for exploring complex phenomena where existing theories may not sufficiently capture the intricacies of the studied context. In this research, we leveraged the principles of grounded theory in two ways: First, during the development of our research model, we drew on existing theories (TAM and IS Success Model), but we allowed the model to be informed and adjusted based on the patterns emerging from the preliminary analysis of the collected data. This iterative process aligns with the constant comparative method of grounded theory, which involves continual refinement of the conceptual framework based on the observed data^100^. Second, grounded theory’s emphasis on understanding the actors’ perspectives was reflected in our analysis of the survey responses. We didn’t merely quantify the responses but sought to understand the underlying reasons for users’ satisfaction or dissatisfaction with the ERP system. This process helped us identify unexpected moderating factors, such as the negative moderation of participation on the perceived usefulness–satisfaction relationship.

The sampling procedure primarily relied on convenience and access to a large-scale manufacturing company, referred to as company “A”, which extensively uses an ERP system in the shipbuilding and marine engineering industries. The decision to focus on this specific company aligns with the rationale provided by Neuman^101^, who suggests that convenience sampling is appropriate when access to a specific population is limited or when other sampling methods are impractical. The selection of this company was motivated by its substantial use of ERP and the diverse user roles involved, which allowed for a comprehensive understanding of user satisfaction. To ensure representativeness within the organization, participants were recruited from various departments and roles, following the principles of stratified sampling as outlined by Creswell and Creswell^102^. This approach involved dividing the population into distinct groups or strata and selecting samples from each stratum to ensure representation. The selected company has a long history of implementing and operating ERP systems since the early 2000s, resulting in a workforce that is familiar with using such systems to streamline operations, manage resources efficiently, and enhance productivity. The survey was distributed randomly to employees across different departments and hierarchical levels, aiming to obtain a diverse cross-section of ERP users within the organization and enhance the validity and reliability of the findings.

We explained the purpose and importance of this study to the company’s executives. Executives agreed to the survey. The in-house enterprise system staffs selected 400 employees using ERP within the company. In order to increase the efficiency of data collection, they produced online questionnaires through groupware and distributed questionnaire links to 400 target employees. A total of 237 data were collected, yielding a response rate of 59.25%. The authors and the staffs conducted a preliminary survey of the collected data to look for any missing information, insincere responses, or outliers. Responses with a high percentage of consistently answering with one answer were removed. A total of 234 replies were deemed to be reliable enough for further examination. The sample size of 234 participants in this study was determined based on the guidelines and recommendations for structural equation modeling (SEM) studies. SEM studies typically require a sufficient sample size to ensure statistical power and reliable estimation of the model parameters^103^. According to Hair et al.^103^, the minimum recommended sample size for SEM studies is 200 participants. This recommendation is based on the need to achieve an acceptable balance between statistical power and the complexity of the model. In studies with a larger number of latent variables and observed variables, a larger sample size is generally required to ensure adequate statistical power^104^. In this study, there are 7 latent variables and 21 observed variables, indicating a moderate level of complexity in the measurement model. While a larger sample size would have been desirable, the sample of 234 participants meets the minimum recommendation for SEM studies and allows for a reasonable estimation of the model parameters^103^. Therefore, considering the guidelines for SEM studies and the demonstrated feasibility of obtaining reliable results with similar sample sizes, the sample of 234 participants in this study is justified.

Table 1 presents the demographic information of the 234 participants in the study. Many participants were male (89.3%), while 10.7% were female. In terms of age distribution, 1.7% of participants were in their 20 s, 41.9% in their 30 s, 41.9% in their 40 s, and 14.5% were in their 50 s. The working period of participants varied, with 9.4% of participants having worked for less than 6 months, 23.9% between 6 months to 1 year, 15.8% between 1 and 2 years, 22.2% between 2 and 5 years, and 28.6% of participants having worked for over 5 years. Regarding the ERP modules used by the participants, 12.0% used Operation Management, 13.7% used Design, 4.7% used Sales/Business Management, 15.0% used Materials/Procurement, 13.7% used Quality Management, 12.8% used Management/Human Resource, 16.7% used Finance Accounting, and 11.5% used other modules. Overall, the sample represents a diverse group of individuals with varying demographic characteristics and differing experiences with various ERP modules.

**Table 1:** Demographic information.

Demographics	Item	Subjects (N = 234)	
		Frequency	Percentage
Gender	Male	209	89.3
	Female	25	10.7
	Total	234	100
Age	20s	4	1.7
	30s	98	41.9
	40s	98	41.9
	50s	34	14.5
	Total	234	100
Working period	Under 6 months	22	9.4
	6 months–1 year	56	23.9
	1–2 years	37	15.8
	2–5 years	52	22.2
	Over 5 years	67	28.6
	Total	234	100
Modules	Operation Management	28	12.0
	Design	32	13.7
	Sales/Business Management	11	4.7
	Materials/Procurement	35	15.0
	Quality Management	32	13.7
	Management/Human Resource	30	12.8
	Finance Accounting	39	16.7
	Others	27	11.5
	Total	234	100

### Ethical approval

This study was approved by an institutional review board of RealSecu.

### Informed consent

Informed consent was obtained from all individual participants included in the study.

### Consent to participate

Consent to participate was obtained from all individual participants included in the study.

## Empirical results

The partial least squares SEM (PLS-SEM) method was employed to estimate the measurement model and the structural model. PLS is the one of types of SEM which is a sophisticated multivariate method^105^. These days, SEM has dominated the research landscape^106^. It has been extensively employed in the organization and IS fields. The hypotheses were tested using SmartPLS 3.0^107^.

This study derived the empirical results through the following analysis steps. It controlled and evaluated the common method bias. Next, this research verified the measurement model and structural model. In the first step, this paper checked and managed the content validity and manage issues related to the common method bias. In the second step, the current study confirmed the reliability and validity of the measurement model. Reliability was confirmed by Cronbach Alpha and composite reliability (CR). This work demonstrated the validity by dividing it into convergence validity and discriminant validity. The convergence validity was confirmed by average variance extracted (AVE), and the discriminant validity was verified by the Fornell and Larcker^108^ criterion. In the second step, the structural model was verified. This study applied a bootstrapping method that generates 5000 resamples. Based on this, the path coefficient and R^2^ values were calculated.

### Common method bias (CMB)

In this study, we took several precautions to mitigate CMB, which can occur when both predictor and criterion variables are obtained from the same source at the same time, potentially inflating the observed relationships^109^. As the data for this study was collected via self-reported surveys, there was a potential risk of CMB. Firstly, we adopted procedural remedies to minimize the chances of CMB. These included assuring the respondents of the confidentiality of their responses and emphasizing that there were no right or wrong answers to the survey questions^109^. This helped reduce evaluation apprehension and encouraged respondents to answer honestly. Secondly, we took steps to separate the measurement of different constructs both in terms of the order of questions and by explicitly signaling to respondents when questions related to different constructs began^109^. This helped reduce the likelihood of participants’ responses to one set of questions influencing their responses to subsequent questions. Finally, we used statistical controls to test for the presence of CMB. Specifically, we conducted a Harman’s single-factor test^110^, where all items are loaded onto a single factor in an exploratory factor analysis. If a single factor emerges or one factor accounts for most of the covariance among the measures, then a substantial amount of CMB is present. In our case, multiple factors emerged and no single factor accounted for most of the covariance, suggesting that CMB was not a major issue in our study. By combining these procedural and statistical controls, we aimed to minimize the risk of common method bias in our study.

### Measurement model

Reliability and validity evaluations were carried out to assess the measurement model. The factor loadings from 0.778 to 0.970 (p = 0.001), indicating strong reliability^111^. Based on Cronbach’s alpha and the composite reliability (CR) value, internal consistency reliability was evaluated. Table 2 shows that all of the constructs have Cronbach’s alpha and CR estimates are more than the recommended minimum value of 0.7^108^, which suggests that adequate reliability exists. By looking at the AVE of the indicators, convergent validity was demonstrated. AVE scores were from 0.723 to 0.929, which are above the cut-off point of 0.5^108^. Finally, to confirm discriminant validity, the root square of AVE values of the each factor were compared to the off-diagonal entries^108^. All of the diagonal entries were over any other corresponding rows or column values, presenting an adequate discriminant validity. Table 3 describes the results of discriminant validity.

**Table 2:** Descriptive statistics and scale reliability.

Construct	Items	Mean	St. Dev.	Factor loading	Cronbach’s Alpha	CR	AVE
Satisfaction	SAT1	4.299	1.395	0.970	0.962	0.975	0.929
	SAT2	4.226	1.407	0.965			
	SAT3	4.252	1.375	0.956			
Perceived ease of use	PEU1	3.688	1.399	0.890	0.838	0.903	0.756
	PEU2	4.226	1.440	0.876			
	PEU3	3.645	1.473	0.842			
Perceived usefulness	PUS1	4.842	1.376	0.945	0.945	0.964	0.900
	PUS2	4.842	1.335	0.957			
	PUS3	4.675	1.407	0.944			
System quality	SYQ1	4.679	1.306	0.889	0.809	0.886	0.723
	SYQ2	4.278	1.373	0.879			
	SYQ3	4.436	1.287	0.778			
Information quality	INQ2	4.859	1.292	0.889	0.845	0.906	0.764
	INQ3	4.675	1.290	0.911			
	INQ4	4.923	1.259	0.818			
Service quality	SEQ1	4.611	1.097	0.903	0.910	0.943	0.847
	SEQ2	4.496	1.167	0.949			
	SEQ3	4.504	1.159	0.909			
Participation	PAT1	4.397	1.274	0.945	0.948	0.966	0.905
	PAT2	4.402	1.343	0.956			
	PAT3	4.436	1.280	0.954

**Table 3:** Results of Fornell–Larcker test.

Construct	1	2	3	4	5	6	7
1. Satisfaction	0.964						
2. Perceived ease of use	0.688	0.869					
3. Perceived usefulness	0.721	0.584	0.949				
4. System quality	0.663	0.515	0.563	0.850			
5. Information quality	0.697	0.559	0.806	0.620	0.874		
6. Service quality	0.667	0.618	0.588	0.519	0.663	0.920	
7. Participation	0.737	0.586	0.670	0.471	0.613	0.610	0.952

### Structural model

**Figure 2:**
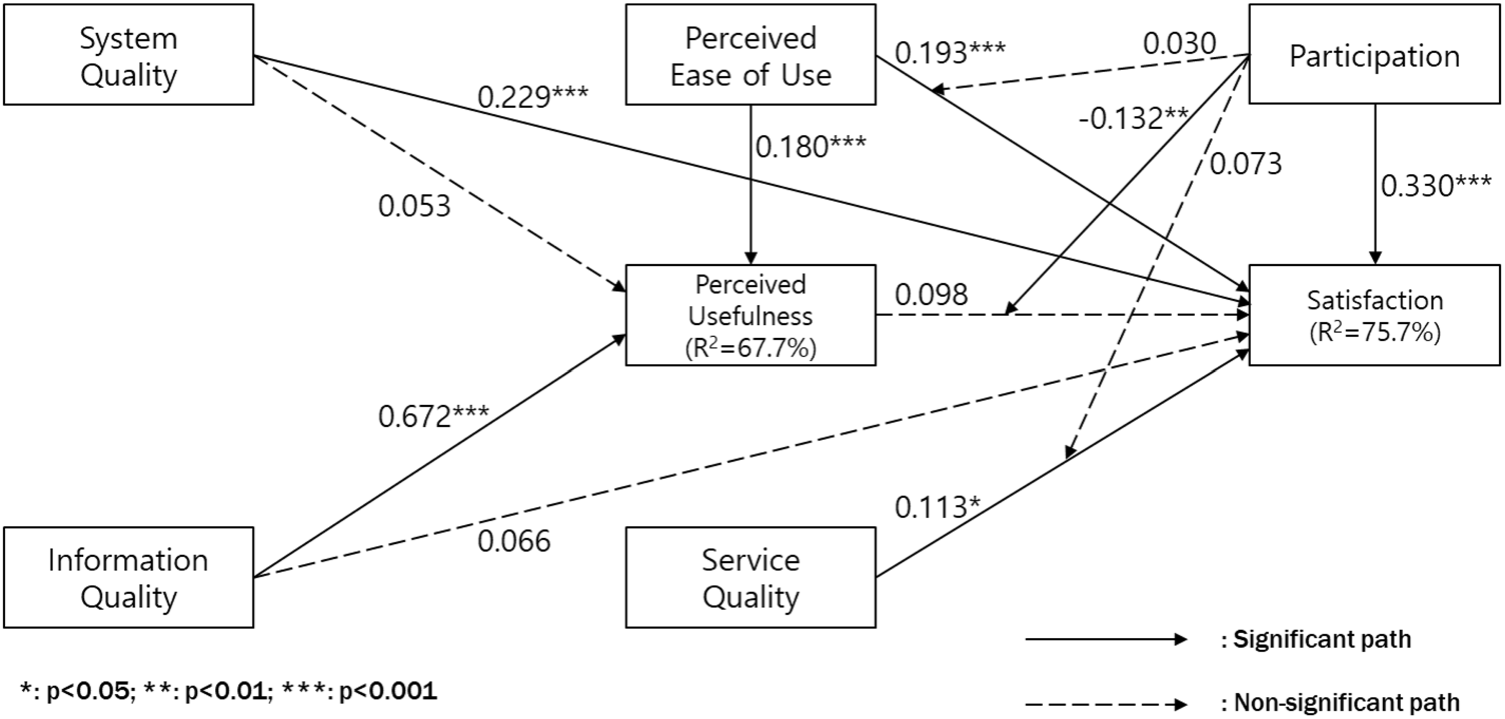
Structural model assessment.

A structural model was analyzed to verify the hypotheses within the model. A bootstrap resampling technique iterated 5000 resamples to calculate the path coefficients and R^2^. The results of structural model are shown in Fig. 2.

Consistent with our expectations, perceived ease of use has a significant impact on both satisfaction and perceived usefulness, providing support for H1a and H1b. However, perceived usefulness does not have a significant effect on satisfaction, resulting in the non-support of H2. System quality has a significant influence on satisfaction but does not affect perceived usefulness, thus accepting H3a and rejecting H3b. Information quality does not directly impact satisfaction, but it strongly influences perceived usefulness, leading to the non-adoption of H4a and the adoption of H4b. Service quality significantly affects satisfaction, offering empirical evidence for H5. As predicted, participation has a strong influence on satisfaction, leading to the acceptance of H6. However, participation does not moderate the relationship between perceived ease of use and satisfaction (H7a), indicating non-support. On the other hand, participation negatively moderates the relationship between perceived usefulness and satisfaction (H7b), supporting this hypothesis. Participation does not moderate the impact of service quality on satisfaction (H7c), failing to accept this hypothesis. The research framework accounts for 75.7% of the variance in satisfaction and 67.7% of the variance in perceived usefulness, as depicted in Table 4, which presents the results structural model assessment.

**Table 4:** Structural model assessment.

H	Cause	Effect	Coefficient	T-value	P-value	Hypothesis
H1a	Perceived ease of use	Satisfaction	0.193	3.515	0.000	Supported
H1b	Perceived ease of use	Perceived usefulness	0.180	3.619	0.000	Supported
H2	Perceived usefulness	Satisfaction	0.098	1.108	0.268	Not supported
H3a	System quality	Satisfaction	0.229	4.277	0.000	Supported
H3b	System quality	Perceived usefulness	0.053	1.046	0.296	Not supported
H4a	Information quality	Satisfaction	0.066	0.830	0.407	Not supported
H4b	Information quality	Perceived usefulness	0.672	12.557	0.000	Supported
H5	Service quality	Satisfaction	0.113	2.133	0.033	Supported
H6	Participation	Satisfaction	0.330	5.434	0.000	Supported
H7a	Participation × perceived ease of use	Satisfaction	0.030	0.486	0.627	Not supported
H7b	Participation × perceived usefulness	Satisfaction	-0.132	3.094	0.002	Supported
H7c	Participation × service quality	Satisfaction	0.073	1.811	0.071	Not supported

## Discussion

The current study explored the predictors of user satisfaction with ERP. It incorporated factors from the IS success model and components from TAM, while also considering participation as an additional variable.

The findings from this study that perceived ease of use influences both satisfaction and perceived usefulness corroborate existing literature within the realm of ERP systems and TAM. The result aligns with the fundamental tenets of TAM, which posits that the perceived ease of use of a technology can affect its perceived usefulness and subsequently the user satisfaction^12^. As well, previous research have validated that perceived ease of use influence satisfaction^64,81,112^ and perceived usefulness^43,113^. The impact of perceived ease of use on satisfaction can be comprehended through the lens of user experience. When users find a system effortless to use, it enhances their interaction with the technology, making it more enjoyable and satisfactory. This outcome resonates with prior research that has established a positive relationship between perceived ease of use and user satisfaction^114,115^. In the context of perceived usefulness, the finding suggests that when users perceive an ERP system to be easy to use, they are more likely to recognize its utility. This is because an easy-to-use system lowers cognitive burden and allows users to focus more on the task at hand rather than on the operation of the system itself, thus enhancing its perceived usefulness^22^.

The finding that perceived usefulness does not significantly affect satisfaction in the context of ERP systems in this study is somewhat surprising, considering the postulates of the TAM and prior research findings^12^. Several previous studies have indicated a positive relationship between perceived usefulness and user satisfaction^80,116,117^. However, this study deviates from those findings, raising several considerations. Firstly, it might be that in the specific context of the study—A big size manufacturing company, a high-level technology environment—other factors could overshadow perceived usefulness in terms of impacting satisfaction. It is plausible that in such a technologically advanced setting, users may take the usefulness of systems for granted, causing other factors to become more influential in determining satisfaction. Secondly, the non-significant relationship might be due to the ERP system’s characteristics or the specific tasks for which it is used. If the tasks are routine or do not require the full functionality of the ERP system, users might not perceive its full usefulness, which could affect their satisfaction. Lastly, there could be measurement or sample-specific factors that led to this unexpected result. It’s possible that the items used to measure perceived usefulness did not capture the construct effectively in this context or for these specific users.

The analysis unveiled that system quality significantly influences satisfaction while it does not affect perceived usefulness. The significant effects of system quality on satisfaction^78,79,118^ and perceived usefulness^80,81^ have been demonstrated in the past research. These findings could be explained by the reason that workers are more satisfied when the ERP system is better structured and provides appropriate functions. The reliability, speed, and understandability of ERP enhance satisfaction, while they can’t guarantee a high level of perceived usefulness.

Although many authors have argued that information quality influences satisfaction^73,79,119^, the empirical findings found that information quality does not impact satisfaction. Several reasons might explain this discrepancy. First, the quality of the information provided by ERP may not be as critical to users as other factors such as system functionality or ease of use. Second, users may have developed strategies to cope with poor information quality, reducing its impact on overall satisfaction. Alternatively, the users’ perceptions of information quality may be influenced by other factors not accounted for in this study. For example, organizational culture, individual user experience, or training may affect how users perceive the quality of information provided by ERP. The analysis showed that information quality strongly affects perceived usefulness, which is consistent with the conclusions of previous studies^64,83,118^. These observations also further support the previous works, which demonstrated the significant relationships between DM success factors and perception components of TAM^8,46,64^. Particularly, information quality is more significant than other factors to let users perceive ERP as useful. This may be because when the information provided by ERP is more understandable and reliable, users perceive it more useful. Since ERP is a company-wide IS, it needs to provide various information according to the needs of users. Because workers use ERP to save time of searching or processing, a higher level of information quality forms a greater degree of perceived usefulness.

The study analysis verified the significance of service quality on satisfaction. This observation further supports the previous research, in which service quality improve satisfaction^79,118,120^. The association between service quality and satisfaction indicates that when the IT support department better solves problems related to ERP, user satisfaction is improved.

The results of this study indicate that user participation significantly impacts satisfaction in the context of ERP. This finding supports the notion that user involvement in IS-related activities, such as system design, implementation, and usage, is crucial in shaping their perceptions and attitudes toward the system^56,59,121^. User participation can manifest in various forms—from providing feedback on system design to active involvement in decision-making processes regarding system implementation and use. This participatory approach can lead to a better understanding of the system’s capabilities, improved alignment with user needs and expectations, and a sense of ownership and commitment to the system, thereby enhancing user satisfaction^26,122^. In the context of ERP, user participation becomes particularly critical given the complexity and organization-wide nature of these systems. ERP systems often require substantial changes in business processes and workflows, which can cause disruption and resistance among users. Active user participation can help mitigate these challenges by fostering better understanding, reducing resistance to change, and enhancing system acceptance and satisfaction^123,124^.

The results of this study suggest that user participation negatively moderates the effects of perceived usefulness on satisfaction in the context of an ERP system. This finding is somewhat unexpected, as prevailing literature generally suggests that user participation has a positive impact on satisfaction^26,59,121^. However, in the case of our study, it seems that increased participation may lessen the impact of perceived usefulness on satisfaction. This surprising result can be understood in several ways. First, it might be that user participation in the context of ERP system use involves a certain degree of cognitive and time investment. The more users participate in system-related activities, the more time and cognitive resources they might need to dedicate to the system. If users perceive the system as useful but find the participation process burdensome or overwhelming, this could negatively affect their satisfaction levels. Second, it’s possible that the quality, rather than the quantity, of user participation is what truly matters. If user participation involves tasks that users find uninteresting, irrelevant, or overly complex, this could lead to frustration or disengagement, thus negatively impacting satisfaction despite high perceptions of usefulness. Last, user participation could create higher expectations for the system’s usefulness. The more users are involved, the more they might expect from the ERP system in terms of its performance and utility. If these heightened expectations are not met, users may become dissatisfied, even if they perceive the system as relatively useful.

The results of this study indicated that user participation did not moderate the effects of perceived ease of use and service quality on satisfaction in the context of an ERP system. This finding might initially appear counterintuitive, given the substantial body of research that suggests user participation plays a pivotal role in enhancing system use and satisfaction^25,26,59^. However, upon closer inspection, this outcome can be interpreted in several ways. First, while participation is often correlated with positive outcomes, its effectiveness may depend on the nature of the system being used. In the case of ERP systems, their complex and integrated nature might mean that simply participating in the system’s use may not be sufficient to improve satisfaction. The user might require a deeper level of engagement or understanding of the system to see improvements in satisfaction. Second, the nature of the tasks performed in an ERP system might play a role. If users are completing complex tasks that require a high level of knowledge and expertise, then merely participating might not lead to increased satisfaction. In such scenarios, the quality of participation, rather than the quantity, might be more critical. Finally, the results might reflect the characteristics of the users themselves. If users have a high level of self-efficacy or prior experience with similar systems, then their satisfaction might be less dependent on their level of participation.

## Conclusion

### Theoretical implications

The current study makes several significant theoretical contributions to the existing body of knowledge, specifically in the realm of ERP systems and user satisfaction. Firstly, by incorporating variables from both the IS success model and the TAM, this research offers a more comprehensive framework for understanding user satisfaction. Although both models have been widely used and validated in numerous studies^12,29^, their combined application in the context of ERP systems remains relatively unexplored. The study’s results shed light on the unique and combined effects of constructs from both models on user satisfaction, thereby enhancing our understanding of the complex dynamics at play. For example, while previous studies have emphasized the role of perceived usefulness and ease of use in user satisfaction^12,115^, our findings underscore the significance of system quality and service quality, which are derived from the IS success model. This suggests that user satisfaction in ERP systems may be influenced by a combination of factors from both models, an aspect that has previously received limited in-depth exploration.

Secondly, this study highlights the role of user participation as a significant predictor of user satisfaction in the context of ERP systems. Despite the extensive research on user participation in IS success^26,59^, its impact in the specific context of ERP systems has received less thorough examination. By demonstrating that user participation significantly influences satisfaction, our study paves the way for more comprehensive models that integrate user participation into these established frameworks. Additionally, our research underscores the complex nature of the relationships among factors influencing ERP satisfaction. For instance, we found that user participation negatively moderates the relationship between perceived usefulness and satisfaction, but not the relationships of perceived ease of use and service quality with satisfaction. This finding signals the need for more intricate models that account for potential moderating or mediating effects among different factors. Furthermore, the contrast between our findings and those from previous studies invites scholars to revisit and refine the measurement of constructs such as perceived usefulness, especially in specific contexts like high-tech manufacturing. Our study suggests that traditional measures of perceived usefulness might not fully capture the construct in this setting, pointing to the need for more context-sensitive measures.

Thirdly, our research challenges the conventional wisdom that perceived usefulness always positively influences satisfaction. While numerous studies have established a positive relationship between perceived usefulness and user satisfaction^12,13,74,115^, our findings reveal that this relationship may not hold true in the context of ERP systems within high-technology environments. This unexpected finding opens new avenues for researchers to explore the conditions under which perceived usefulness may or may not impact satisfaction. It suggests that the impact of perceived usefulness on satisfaction may be contingent upon various factors, such as the nature of the tasks, specific system features, or user characteristics, thereby calling for a more nuanced understanding of the relationship between perceived usefulness and satisfaction.

Finally, the unique features of our study sample provide additional theoretical contributions. Specifically, our study was conducted within a large-scale manufacturing company operating in a high-technology environment. This context has its characteristics that add new insights to the existing body of knowledge on ERP satisfaction. Previous research has largely focused on more general or diverse settings, failing to examine how the dynamics within high-tech, manufacturing environments might impact user satisfaction with ERP systems^62,125,126^. In our study, the finding that perceived usefulness does not significantly affect satisfaction is particularly noteworthy, as it contradicts the typical assumptions of the TAM and previous empirical findings. This unexpected result suggests that in high-tech environments, where the utility of systems is often taken for granted, other factors might become more critical in determining satisfaction. This could be the case in large-scale manufacturing companies where technology application is widespread and advanced, and users might have a higher baseline expectation for system usefulness. Additionally, our study revealed that user participation has varying effects on satisfaction and its predictors, which further extends the understanding of the user participation role in the IS success literature. Particularly, in a high-tech manufacturing setting, users might be more specialized and experienced^127^, leading to different dynamics between participation, perceived usefulness, and satisfaction compared to more general settings. These findings offer a more nuanced view of ERP satisfaction determinants, particularly in high-tech manufacturing settings. They suggest the need for further research to examine how specific organizational contexts, such as industry type, technology maturity, or user characteristics, might impact the relationships among factors influencing ERP satisfaction. This context-specific approach can yield more granular insights, enhancing the theoretical understanding of ERP satisfaction and providing more precise guidance for practice.

### Practical implications

The results of this study offer several practical implications for organizations implementing ERP systems. The first significant implication is the importance of system quality and service quality in enhancing user satisfaction. Given that our findings show a strong positive correlation between these constructs and user satisfaction, organizations must pay careful attention to the design and ongoing maintenance of their ERP systems. For instance, they need to ensure that the system is reliable, efficient, and user-friendly to enhance its perceived quality. Likewise, the quality of IT support services is crucial. Organizations need to establish efficient helpdesk services, regular system checks, and prompt troubleshooting mechanisms to resolve user issues swiftly. Training programs aimed at enhancing users’ skills in using the system can also help boost service quality.

Secondly, our findings underscore the role of user participation in shaping satisfaction with ERP systems. This suggests that organizations need to actively involve end-users in the process of ERP system design, implementation, and use. For example, organizations could include representatives from different user groups in the system design and selection process to ensure that the chosen system meets users’ needs and preferences. Similarly, encouraging user involvement in decision-making related to the system’s use, such as customizing system features or defining process workflows, can enhance their sense of ownership and commitment to the system, thereby boosting satisfaction. Organizations can also establish feedback mechanisms to solicit users’ opinions and suggestions about the system, thereby enabling continuous improvement based on user input.

Thirdly, the fact that perceived usefulness does not significantly affect satisfaction in our study implies that organizations should not solely rely on promoting the usefulness of ERP systems to enhance user satisfaction. While demonstrating the system’s utility is undoubtedly important, it might not be sufficient in high-tech environments where users might take the system’s usefulness for granted. Instead, organizations should focus on other aspects, such as providing a seamless user experience, ensuring system reliability, and offering efficient user support services. For instance, in a manufacturing context, ensuring that the ERP system integrates well with production workflows and provides real-time, accurate information could be more crucial than simply showcasing the system’s functionality.

Fourthly, our study’s results suggest that user participation does not necessarily amplify the effects of perceived ease of use and service quality on satisfaction. This means that organizations should not merely focus on increasing user participation in the hope of improving satisfaction. Instead, they need to consider the nature of the tasks performed in the ERP system and the characteristics of the users. For example, in situations where users perform complex tasks requiring specialized knowledge, organizations might need to invest in more intensive training or provide additional support to enhance satisfaction. Similarly, if users have high self-efficacy or prior experience with similar systems, their satisfaction might be more influenced by factors such as system performance, information quality, or the responsiveness of support services.

Lastly, our findings highlight the importance of information quality in influencing perceived usefulness, although it does not directly impact satisfaction. This implies that organizations should focus on improving the quality of information provided by ERP systems to enhance users’ perceived usefulness, even if it may not directly boost satisfaction. This can be achieved by ensuring data accuracy, timeliness, completeness, and relevancy. For instance, organizations could implement robust data governance practices, automate data input processes to reduce errors, and regularly audit the system data to ensure its quality. By enhancing the perceived usefulness of the system through improved information quality, organizations can indirectly contribute to overall user satisfaction.

### Limitations and future research

The current paper has several limitations. Firstly, the data collected for this study was limited to South Korea, which may limit the generalizability of the findings to other countries with different business environments and industries. Future research should consider surveying multiple countries to enhance the external validity of the study. Secondly, this paper only considered participation as a moderating variable, neglecting other potential variables such as commitment to learning, peer impact, and top manager engagement. Including these variables in future studies would provide a more comprehensive understanding of the research topic. Additionally, future research could introduce additional control variables to enhance the explanatory power of the proposed research model. Thirdly, this study focused exclusively on ERP systems, and it would be beneficial to apply the theoretical framework to other enterprise information systems like supply chain management and customer relationship management for comparative analysis. By doing so, the reliability of the research model can be further established. Moreover, factors such as training and support, as well as financial outcomes and operational efficiency, were not comprehensively included as exogenous and final variables, respectively. Future research should strive for a balanced inclusion of various variables to generalize the results more effectively. Lastly, the study is limited by its small sample size, which may impact the generalizability and robustness of the conclusions. Future research should aim to include a larger and more diverse sample to enhance the generalizability and strengthen the validity of the study’s findings.

### Supplementary Information

Supplementary Information.

## Data Availability

The datasets used and/or analyzed during the current study available from the corresponding author on reasonable request.
