# Supplementary material for: Mechanisms for successful management of enterprise resource planning from user information processing and system quality perspective
Source: Sci Rep. 2023 Aug 4;13:12678. doi: 10.1038/s41598-023-39787-y (PMC10403517; doi:10.1038/s41598-023-39787-y)
Supplement: Supplementary file 1 — Supplementary Information. [file 41598_2023_39787_MOESM1_ESM.docx]

# Appendix A. Lists of Measurement Items

Table S1. Indicators of Constructs and Sources

| Construct | Items | Mean | Source |
| --- | --- | --- | --- |
| Satisfaction | SAT1 | The ERP satisfies me on the whole. | [Costa et al. (2016)](#_ENREF_21) |
|  | SAT2 | The ERP is efficient. |  |
|  | SAT3 | The ERP is effective. |  |
| Perceived Ease  of Use | PEU1 | I find it easy to get the ERP to do what I want it to do. | [Costa et al. (2016)](#_ENREF_21) |
|  | PEU2 | It is easy for me to remember how to perform tasks using the ERP. |  |
|  | PEU3 | Overall, I find the ERP easy to use. |  |
| Perceived  Usefulness | PUS1 | ERP enables me to accomplish tasks more quickly. | [Costa et al. (2016)](#_ENREF_21) |
|  | PUS2 | Using ERP increases my productivity. |  |
|  | PUS3 | Using ERP improves the quality of the work I do. |  |
| System  Quality | SYQ1 | ERP is well structured. | [Costa et al. (2016)](#_ENREF_21) |
|  | SYQ2 | ERP allows me to easily find the information I am looking for. |  |
|  | SYQ3 | ERP offers appropriate functionality. |  |
| Information  Quality | INQ1 | The information provided by ERP is understandable. | [Urbach and Müller (2012)](#_ENREF_82) |
|  | INQ2 | The information provided by ERP is up-to-date. |  |
|  | INQ3 | The information provided by ERP is reliable. |  |
| Service  Quality | SEQ1 | The responsible service personnel have sufficient knowledge to answer my questions in respect of the ERP. | [Urbach and Müller (2012)](#_ENREF_82) |
|  | SEQ2 | The responsible service personnel provide personal attention when I experience problems with the ERP. |  |
|  | SEQ3 | The responsible service personnel provide services related to the ERP at the promised time. |  |
| Participation | PAT1 | I am committed to the success of ERP. | [Barki and Hartwick (1994)](#_ENREF_13) |
|  | PAT2 | I participate in the development and evaluation of ERP. |  |
|  | PAT3 | I suggest/review periodic enhancements of ERP. |  |
